# Short-Term Exposure of Paddy Soil Microbial Communities to Salt Stress Triggers Different Transcriptional Responses of Key Taxonomic Groups

**DOI:** 10.3389/fmicb.2017.00400

**Published:** 2017-03-28

**Authors:** Jingjing Peng, Carl-Eric Wegner, Werner Liesack

**Affiliations:** Department of Biogeochemistry, Max Planck Institute for Terrestrial MicrobiologyMarburg, Germany

**Keywords:** salt stress, metatranscriptomics, mRNA, methanogenesis, methane, Clostridiaceae, Methanosarcinaceae, Methanocellaceae

## Abstract

Soil salinization due to seawater intrusion along coastal areas is an increasing threat to rice cultivation worldwide. While the detrimental impact on rice growth and yield has been thoroughly studied, little is known about how severe salinity affects structure and function of paddy soil microbial communities. Here, we examined their short-term responses to half- and full-strength seawater salinity in controlled laboratory experiments. Slurry microcosms were incubated under anoxic conditions, with rice straw added as carbon source. Stress exposure time was for 2 days after a pre-incubation period of 7 days. Relative to the control, moderate (300 mM NaCl) and high (600 mM NaCl) salt stress suppressed both net consumption of acetate and methane production by 50% and 70%, respectively. Correspondingly, community-wide mRNA expression decreased by 50–65%, with significant changes in relative transcript abundance of family-level groups. mRNA turnover was clearly more responsive to salt stress than rRNA dynamics. Among bacteria, Clostridiaceae were most abundant and the only group whose transcriptional activity was strongly stimulated at 600 mM NaCl. In particular, clostridial mRNA involved in transcription/translation, fermentation, uptake and biosynthesis of compatible solutes, and flagellar motility was significantly enriched in response salt stress. None of the other bacterial groups were able to compete at 600 mM NaCl. Their responses to 300 mM NaCl were more diverse. Lachnospiraceae increased, Ruminococcaceae maintained, and Peptococcaceae, Veillonellaceae, and Syntrophomonadaceae decreased in relative mRNA abundance. Among methanogens, Methanosarcinaceae were most dominant. Relative to other family-level groups, salt stress induced a significant enrichment of transcripts related to the CO dehydrogenase/acetyl-coenzyme A synthase complex, methanogenesis, heat shock, ammonium uptake, and thermosomes, but the absolute abundance of methanosarcinal mRNA decreased. Most strikingly, the transcriptional activity of the Methanocellaceae was completely suppressed already at 300 mM NaCl. Apparently, the key taxonomic groups involved in the methanogenic breakdown of plant polymers significantly differ in their ability to cope with severe salt stress. Presumably, this different ability is directly linked to differences in their genetic potential and metabolic flexibility to reassign available energy resources for cellular adaptation to salt stress.

## Introduction

Increased salinization of arable land is expected to have devastating global effects, resulting in up to 50% land loss by the year 2050 (Wang et al., 2003). High levels of salinity provoke the withering of plants as a result of both the increase in osmotic pressure and the toxic effects of salts (Gupta and Huang, 2014). A particular threat to worldwide crop production is seawater intrusion along inland deltas and coastal areas. Here, rice farming is at high risk, considering that growing rice consumes up to two or three times more water per hectare than other crop (Tuong et al., 2005; Williams, 2010). The effects of seawater intrusion on rice growth and yield, including possible management and combat strategies, have therefore been thoroughly studied (Supparattanapan et al., 2009; Nautiyal et al., 2013). Seawater intrusion greatly varies in time and space, but events of saltwater intrusion are predicted to increase strongly in both frequency and magnitude due to climate change and sea-level rise. This will affect more and more rice acreage that has never been exposed to high salinity (Dasgupta et al., 2009, 2014; Vermeer and Rahmstorf, 2009; Hoggart et al., 2014). One liter of seawater contains approximately 35 g of dissolved salts with sodium chloride (NaCl) as the predominant component. Transient exposure of paddy soils to NaCl can be as high as half- to full-strength seawater salinity (Kotera et al., 2008).

On a global scale, salinity has been shown to be the major environmental determinant of microbial community composition (Lozupone and Knight, 2007). However, little is known about how seawater salinity affects the structure and function of paddy soil microbial communities, despite the fact that soil microorganisms are crucial for nutrient cycling, soil fertility, and crop productivity (Luo et al., 2016). While only a single study has examined the effects of salinity on methane emission from flooded rice fields (van der Gon and Neue, 1995), some research has been performed to investigate the impact of different levels of seawater salinity on the biogeochemistry (Chambers et al., 2011; van Dijk et al., 2015) and microbial community in soils from natural freshwater wetlands (Baldwin et al., 2006; Morrissey and Franklin, 2015; Nelson et al., 2015). These wetland soils contain highly diverse bacterial communities that are dominated by Proteobacteria, Chloroflexi, Acidobacteria, Nitrospirae, Bacteroidetes, and Verrucomicrobia (Morrissey and Franklin, 2015). Increasing salinity had no or only minor effects on the community composition, even if the salt treatments were extended to one month (Baldwin et al., 2006; Nelson et al., 2015). These studies, however, were performed only on DNA level, using PCR in combination with either sequencing or community fingerprinting of 16S rRNA genes.

Here we examined the short-term responses of paddy soil microbial communities to half- and full-strength seawater salinity on both rRNA and mRNA level. The research was performed in controlled laboratory experiments, with rice straw added as carbon source. Slurry microcosms were incubated under anoxic conditions, given that rice cultivation mainly occurs under flooded conditions (FAO, 2004). Exposure to salt stress was for 2 days after a pre-incubation period of 7 days. These slurry microcosms have been proven to be highly valuable in elucidating the metabolic processes and microbial community dynamics during the methanogenic breakdown of plant polymers (Weber et al., 2001a,b; Wegner and Liesack, 2016). Rice straw and other organic matter is commonly used as a fertilizer, but also frequently added to saline paddy soils to reduce the adverse effects of salinization on rice productivity (Supparattanapan et al., 2009). Methanogenic decomposition of rice straw determines the composition of a complex microbial community that is highly dominated by Firmicutes (Clostridiaceae, Lachnospiraceae, Ruminococcaceae) and methanogenic archaea (Euryarchaeota) (Peng et al., 2008; Rui et al., 2009; Conrad et al., 2012; Wegner and Liesack, 2016). Microbial communities in straw-amended paddy soils thus differ in their composition from those in freshwater wetland soils, with methanogens as a common component.

The major objective of our study was to assess the potential of rRNA- and mRNA-based metatranscriptomics for high-resolution analysis of the community responses to increasing salinity. More specifically, we aimed to elucidate the transcriptome responses of key taxonomic groups involved in the methanogenic polymer breakdown, with a particular focus on their functional capability to cope with moderate and high salt stress.

## Materials and Methods

### Paddy Soil Slurries and Sampling

Soil was collected from drained rice fields at the Italian Rice Research Institute (IRRI) located in the valley of the Po river (Vercelli, Italy). Slurry microcosms were set up by filling 125 mL bottles with 40 g dry soil and 35 mL of autoclaved water. A total of 0.5 g rice straw (*Oryza sativa* var. Roma) pieces (1–2 cm) was added as the main carbon source (e.g., Conrad and Klose, 1999, 2006; Wegner and Liesack, 2016). The carbon–nitrogen (C/N) ratio of rice straw is in the range of 50:1 to 60:1, with a carbon content of 30–40% and a nitrogen content of 0.6–0.7% (Maramba, 1978; Wang et al., 2014). The thoroughly mixed slurries were sealed with butyl rubber stoppers and repeatedly flushed with N_2_ to establish anoxic conditions. Prior to the NaCl treatments, slurries were pre-incubated for 7 days in the dark at 30°C. Then, NaCl was dissolved in 5 mL autoclaved water to adjust slurries to a concentration of 0, 300, and 600 mM NaCl corresponding to 0, 17.5, and 35 g/L. The slurries were shaken vigorously and then incubated for 2 days without shaking. After the NaCl treatment, the unstirred 2-cm water layer and the rice straw were withdrawn and discarded. The slurry material beneath the top layer was collected for molecular analysis and immediately shock-frozen using liquid nitrogen and then kept at -80°C until further processing. Gas samples (0.2 mL) and liquid samples (0.5 mL) were taken from each slurry microcosm at three time points for process measurements: 0 and 7 days after flooding (pre-incubation period); and 2 days after the addition of NaCl. Molecular analyses were performed for control (0 mM NaCl) and the two salt treatments (300 and 600 mM NaCl) after a total incubation period of 9 days (7-day pre-incubation followed by 2-day stress exposure). Independent analysis of triplicate slurries for each experimental treatment included process measurements, qPCR and RT-qPCR, as well as metatranscriptomic analysis of both 16S rRNA and enriched mRNA. Taxonomic profiling of 16S rRNA was based on metatranscriptomic analysis of total RNA. Given the relatively low amounts of mRNA in total RNA (see below), stress-induced changes in the taxonomic and functional mRNA profiles, however, were assessed using enriched mRNA.

### Process Measurements

Methane production was determined by analysis of headspace samples using a gas chromatograph equipped with a Sephadex^®^ column and a methanizer (Conrad and Klose, 1999). The availability of acetate was monitored by ion exclusion HPLC (Conrad and Klose, 2006) applying an Aminex^®^ HPX87H organic acid analysis column (Bio-Rad, Munich, Germany). Obtained data were analyzed using PeakSimple (SRI Instruments, Bad Honnef, Germany) and Clarity (DataApex, Prague, Czechia).

### Nucleic Acid Extraction

Total RNA and enriched mRNA were extracted from slurries using a previously described method (Mettel et al., 2010). Six aliquots (6 × 0.5 g of soil) were extracted from each replicate slurry for analysis of total RNA. The six RNA extracts were pooled, treated with DNase I (Ambion, Austin, TX, USA), and purified using the RNA Clean and Concentrator kit (Zymo Research, Irvine, CA, USA) according to the manufacturer’s instructions. Enriched mRNA was obtained by subjecting total RNA (>2.5 μg) to subtractive hybridization using the Ribo-Zero^TM^ Magnetic Kit for Bacteria (Epicentre, Madison, WI, USA). Generally, 16 extracts of total RNA (each from 0.5 g of soil) were obtained from each replicate slurry and purified using the RNA Clean and Concentrator kit (Zymo Research) according to the manufacturer’s instructions. These 16 RNA extracts were pooled prior to quantitative removal of rRNA by subtractive hybridization. Quality and integrity of the purified RNA and enriched mRNA were checked by Bio-Rad Experion^TM^ and RNA HighSens Chips (Bio-Rad, Hercules, CA, USA). Genomic DNA was extracted using the FastDNA^®^ SPIN kit for soil (MP Biomedicals, Eschwege, Germany).

### Quantitative PCR and RT-PCR

Bacterial and archaeal 16S rRNA gene and transcript numbers per gram of dry soil were determined by qPCR and RT-qPCR, respectively (Wegner and Liesack, 2016). In addition, numbers of genes and transcripts encoding a key enzyme of methanogens, methyl coenzyme reductase A (*mcrA*), were quantified. Total RNA was randomly reverse transcribed using the GoScript Reverse Transcription System (Promega, Mannheim, Germany). Standard curves were constructed using genomic DNA from *Escherichia coli* (bacterial 16S rRNA genes/transcripts, calibration range from 10 to 10^8^ copies) and PCR amplicons (archaeal 16S rRNA genes/transcripts, calibration range from 10 to 10^8^ copies; and *mcrA* genes/transcripts, calibration range from 10 to 10^9^ copies). The analyses were performed using SYBR Green-based assays (Stubner, 2004; Kemnitz et al., 2005; Angel and Conrad, 2013). qPCR was carried out using a CFX Connect Real-Time PCR detection system (Bio-Rad). All reactions were carried out in three technical replicates. The PCR efficiency was at least 85% (*R*^2^ > 0.99). The absence of non-specific products was confirmed by melt curve analysis.

### Illumina RNA-Seq

In total, nine samples each from total RNA and enriched mRNA were subjected to cDNA synthesis using the NEBNext^®^ Ultra^TM^ Directional RNA Library Prep Kit for Illumina^®^ (New England Biolabs, Ipswich, MA, USA) according to the manufacturer’s instructions. cDNA yield and quality were determined using DNA HighSens Chips (Bio-Rad). The 18 cDNA libraries were sequenced at the Max Planck Genome Centre Cologne using 250-bp Illumina MiSeq.

### Bioinformatic Analyses

The raw Illumina paired-end reads were trimmed by removal of residual adaptor sequences using Cutadapt (Martin, 2011) and filtered using a minimum mean quality score of 20. 16S rRNA reads were extracted from total RNA datasets by SortMeRNA 2.0 (Kopylova et al., 2012). For enriched mRNA, reads mapping to rRNA and non-coding small RNA were filtered out by SortMeRNA 2.0, using SILVA and RFAM as reference databases. The remaining reads were considered to be mRNA.

Analysis of 16S rRNA (total RNA) was carried with a customized pipeline that applies USEARCH (Edgar, 2013) and QIIME (Caporaso et al., 2010) as overall framework. OTU clustering was done using the UCLUST algorithm integrated in USEARCH and an OTU threshold of 95%. Using a custom PYTHON script, the resulting OTU table was converted to match the particular specifications of QIIME. Taxonomic assignments to the OTU table were carried out using the RDP classifier method (Wang et al., 2007) implemented in QIIME with SILVA SSU (release: 119; Quast et al., 2013) as the reference database. A total of 5,993,643 quality-filtered reads were obtained from total RNA, of which 3,098,833 reads were derived from 16S rRNA (Supplementary Table S1).

Taxonomic assignment and functional annotation of enriched mRNA was carried using the UBLAST algorithm implemented in USEARCH applying an *e*-value cutoff of 1e-5 for database searches against NCBI’s non-redundant protein database (Pruitt et al., 2012). MEGAN5 (Huson et al., 2011) was used for parsing and downstream analysis of the UBLAST output. Gene information identifier mapping and tabular BLAST output files were loaded to MEGAN. Transcripts that had a hit in the NCBI’s nr protein database were functionally classified into SEED subsystems. A total of 1,607,909 reads had a taxonomic hit in this database (Supplementary Table S2).

### Statistical Analysis

Significant (*P* < 0.05) differences in process measurements and RT-qPCR data between control (0 mM NaCl) and the NaCl treatments (300 and 600 mM NaCl) were determined by analysis of variance (ANOVA). Statistical Analysis of Metagenomic Profiles (STAMP 2.0.8; Parks et al., 2014) was used to identify biologically relevant differences within the (i) 16S rRNA and (ii) mRNA datasets. Significant (*P* < 0.05) differences were determined by ANOVA and subsequently corrected for multiple tests using Benjamini–Hochberg false discovery rate (*Q*-value; Benjamini et al., 2001).

### Data Deposition

The sequence data have been submitted to GenBank under ID BioProject numbers PRJNA313183 (total RNA) and PRJNA302859 (enriched mRNA). This involves the accession numbers SRP077890 (total RNA) and SRP066471 (enriched mRNA).

## Results and Discussion

Amendment of rice straw strongly stimulated microbial activity during the 7-day pre-incubation period, as evidenced by high bacterial and archaeal 16S rRNA transcript:gene ratios (around 100, Supplementary Figure S1). Following the pre-incubation period, we assessed the effect of 6-hour and 2-day stress exposure on the process activities. Six-hour stress exposure, however, had no significant effect on acetate turnover or methane production. Therefore, our research was fully focused on 2-day exposure to salt stress. In pure-culture transcriptome studies, this exposure time is considered long-term incubation given that microorganisms that are able to cope with moderate and high salt stress exhibit an adaptive transcriptome response within 2 days (Zaprasis et al., 2015; Nagler et al., 2016).

### Process Measurements

**FIGURE 1:**
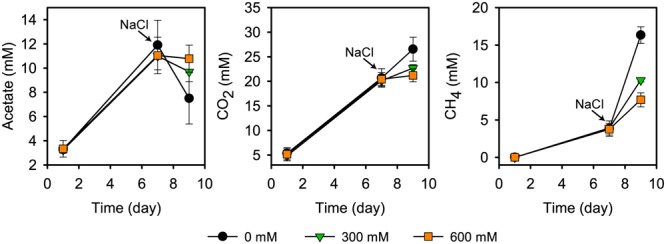
**Acetate concentration, CO_2_ and CH_4_ production prior to and after addition of NaCl (means ± SE; *n* = 3).** NaCl was added after 7 days of pre-incubation. Salt exposure time was 2 days.

Acetate started to accumulate at pretreatment day 1 and reached its transient peak concentration of 11.9 ± 2.3 mM at pretreatment day 7. In the control treatments, the time period between days 7 and 9 was defined by high CH_4_ production (from 3.8 ± 1.0 to 16.3 ± 1.1 mM), in correspondence to a strong decline in the acetate concentration (from 11.9 ± 2.3 to 5.2 ± 0.6 mM) (**Figure 1**). This is in good agreement with previous research (Wegner and Liesack, 2016). Salt stress inhibited CH_4_ production, thereby leading to an accumulation of acetate. There was a significant correspondence between the inhibition effect and severity of salt stress (*P* < 0.01; **Figure 1**). Salt stress reduced net consumption of acetate by 55.3% (300 mM NaCl) and 70.9% (600 mM NaCl). Correspondingly, methane production was reduced by 48.9% and 68.5%, respectively. The corresponding values for reduced CO_2_ production were 56.5% and 82.4%.

The strong inhibition of CH_4_ production agrees well with previous results about how salinization affects methanogenic communities in natural freshwater wetlands (Baldwin et al., 2006; Chambers et al., 2011; van Dijk et al., 2015) and paddy soils (van der Gon and Neue, 1995). Overall, these studies also observed a negative correlation between salinity and methane production. Similar to our results, the incubation of freshwater wetland soil with full-strength seawater salinity (35 g/L NaCl) led, relative to freshwater controls, to an inhibition of CH_4_ production by approximately 70% within the first few days of incubation (Chambers et al., 2011).

### qPCR and RT-qPCR

Salt stress induced a significant decrease (*P* < 0.05) in the 16S rRNA gene and transcript numbers of both bacteria and archaea between control and 600 mM NaCl. The decrease was in the range of up to half an order of magnitude. Stress also induced a significant decrease in the *mcrA* gene and transcript numbers. In 600 mM NaCl, copy numbers were decreased (*P* < 0.05) by 34% (gene) and 59% (transcripts) relative to the control (Supplementary Figure S1).

### mRNA Proportion in Total RNA

The percentage proportion of mRNA in total RNA significantly decreased (*P* < 0.005) with increasing salt stress, from 4.22 ± 1.15% (control) to 2.15 ± 0.23% (300 mM NaCl) and 1.54 ± 0.11% (600 mM NaCl) (Supplementary Table S1). Relative to the control (100%), the mRNA proportion thus decreased by ∼50% and ∼65%, respectively.

### Inhibition of Metabolic and Transcriptional Activity

The stress-induced decline in process activities corresponds well to the decrease in absolute transcript abundance (16S rRNA, *mcrA*) but, even more pronounced, to the decrease in the mRNA proportion in total RNA. Apparently, this proportion is an excellent proxy for changes in the community-wide expression level of functional genes. As such, it also seems to be a suitable molecular indicator for changes in process activities. There was a nearly exact correspondence between the stress-induced decrease in net acetate consumption, methane production, and the proportion of mRNA in total RNA [∼50% (300 mM NaCl) and 65–70% (600 mM NaCl)]. Obviously, mRNA turnover is more responsive to environmental change and adverse conditions than rRNA dynamics, in good correspondence to the mRNA half-lives that are known to be for bacteria in the order of minutes and for methanogenic archaea in the range of tens of minutes and a few hours (Evguenieva-Hackenberg and Klug, 2009; Peterson et al., 2016). The strong decrease in functional gene expression, however, was related to tremendous changes in the taxonomic and functional profiles of enriched mRNA.

### Stress-Induced Changes in Taxonomic Profiles

**FIGURE 2:**
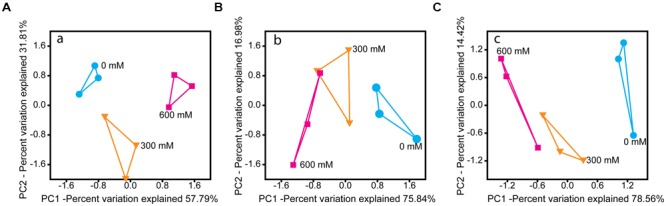
**PCA of the rRNA- (A)** and mRNA-centered **(B,C)** metatranscriptome data. PCA plots were calculated based on the taxon-specific composition of metatranscriptomic 16S rRNA **(A)** and mRNA **(B)**, using the relative abundance values of archaeal and bacterial families. In panel **(C)**, the PCA plot was calculated based on the functional profiles on SEED level 1 subsystems (Supplementary Figure S4).

Salt stress induced significant changes in the microbial community metatranscriptome of total RNA (16S rRNA) and enriched mRNA (**Figures 2A,B**). Except Chloroflexi, all phylum-level groups showed significant changes in their relative rRNA abundance. Acidobacteria and Planctomycetes were the only phylum-level groups whose relative mRNA abundance decreased significantly by 77.0% (*P* < 0.001) and 51.0% (*P* < 0.01), respectively (Supplementary Figure S2).

**FIGURE 3:**
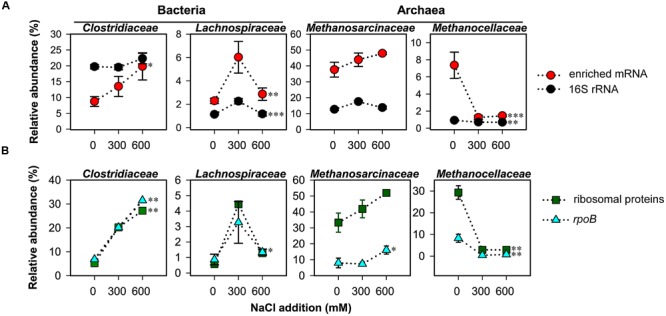
**Changes in taxon-specific transcript abundance of 16S rRNA and enriched mRNA (A)**, and *rpoB* and ribosomal proteins **(B)** after 2-day stress exposure (means ± SE, *n* = 3). Changes are shown for key taxonomic groups. See Supplementary Tables S1, S2 for sequencing statistics of 16S rRNA and taxonomically assigned mRNA, respectively. Broken lines are added only for illustrative purposes. Significance of stress-induced changes is indicated by ^∗^*P* < 0.05, ^∗∗^*P* < 0.01, and ^∗∗∗^*P* < 0.001.

On family level, changes in relative taxon abundance were greater for 600 mM NaCl than for 300 mM NaCl stress exposure and clearly more pronounced in mRNA than rRNA analysis (**Figure 3A** and Supplementary Figure S3). The stress-induced response, however, greatly varied between the different family-level groups. Among bacteria, Clostridiaceae were most competitive and the only bacterial group that significantly increased in relative mRNA abundance, from 8.7% (control) to 19.8% (600 mM NaCl; *P* < 0.05). By contrast, their contribution to total 16S rRNA did not significantly change. Lachnospiraceae were able to combat salt stress at 300 mM NaCl. Their relative mRNA abundance increased significantly (*P* < 0.01), but declined when exposed to 600 mM NaCl (**Figure 3A**). The Peptococcaceae, Veillonellaceae, and Syntrophomonadaceae were unable to compete, even at 300 mM NaCl. The relative mRNA abundance of the Ruminococcaceae was fairly stable at 300 mM NaCl, but significantly decreased at 600 mM NaCl. Bacillaceae showed no significant abundance change, neither on rRNA nor on mRNA level (Supplementary Figure S3).

Among the methanogenic archaea, Methanosarcinaceae showed a highly competitive response to salt stress relative to other family-level groups. Their relative rRNA (15.9–17.7%) and mRNA (37.6–47.9%) abundances were by trend, but not significantly, increased in response to salt stress (**Figure 3A**). The Methanosarcinaceae are known to harbor halotolerant and halophilic members (Kendall and Boone, 2006) and to outcompete *Methanosaeta* spp. at high acetate concentrations. The ability to respond quickly with physiological activity and growth to increased acetate levels is linked to three *rrn* gene copies in all *Methanosarcina* spp. versus one or two in *Methanosaeta* spp. (Stoddard et al., 2015). The early stages of rice straw decomposition are characterized by a rapid but transient increase in acetate levels (**Figure 1**). Thus, the genetic and metabolic potential coupled with high substrate availability may be the major reason why the Methanosarcinaceae were able to respond to severe salt stress more effectively than most other family-level groups and to maintain their relative dominance in the community-wide mRNA pool (**Figure 3A**).

The most striking effect of salt stress was observed for the Methanocellaceae. While their contribution to total 16S rRNA only decreased from 1.1% (control) to 0.7% (600 mM NaCl), their relative mRNA abundance strongly declined from 7.4% (control) to 1.3% already when exposed to 300 mM NaCl (**Figure 3A**). Their unability to cope with salt stress agrees well with the low salt tolerance of taxonomically described *Methanocella* spp.: 17 mM NaCl (*M. paludicola*; Sakai et al., 2008); 86 mM NaCl (*M. conradii*; Lü and Lu, 2012); and 340 mM NaCl (*M. avoryzae*; Sakai et al., 2010). *M. avoryzae* would be able to tolerate 300 mM NaCl but has a temperature growth range of 37–55°C. Significant changes on both rRNA and mRNA level were also evident for the Methanosaetaceae and Methanobacteriaceae (Supplementary Figure S3).

The bacterial single-copy gene encoding RNA polymerase β subunit (*rpoB*) was shown to provide a phylogenetic resolution comparable to or even higher than the bacterial 16S rRNA gene (Case et al., 2007; Vos et al., 2012). We extracted the total number (2,247) of bacterial *rpoB* transcripts and archaeal homologs from the mRNA datasets obtained for control and NaCl treatments. The taxon-specific changes in *rpoB* abundance of the Clostridiaceae, Lachnospiraceae, Methansarcinaceae (at 600 mM NaCl), and Methanocellaceae were highly consistent with those observed for these families in total mRNA. The exactly same trend in taxon-specific abundance changes was also evident for mRNA encoding ribosomal proteins (**Figure 3B**).

### Stress-Induced Changes in Functional Profiles

**FIGURE 4:**
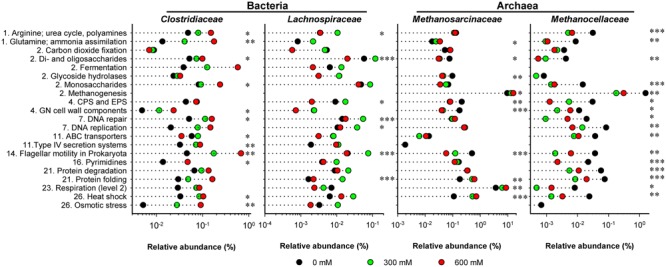
**Stress-induced changes in the functional profiles shown for key taxonomic groups.** These profiles are based on the 21 SEED level 2 categories that showed most significant changes (*P* < 0.01) in community-wide analysis (Supplementary Figure S5). The mRNA reads affiliated with these 21 categories were extracted from the total metatranscriptomes and blasted against the NCBI nr database using the UBLAST algorithm. An e-value cutoff of 1e-5 was applied. The BLAST output was uploaded to MEGAN for analysis of stress-induced changes in taxonomic composition. This analysis was carried out for each of the 21 SEED level 2 categories separately. The family-specific mRNA abundances were calculated as percentages of the total mRNA reads that could be assigned on SEED levels 2–4 (Supplementary Table S2). Numbering of categories relates to the SEED level 1 categories as shown in Supplementary Figure S4. CPS and EPS mean “Capsular and extracellular polysaccharides” and GN means “Gram-negative”. Note that changes in relative mRNA abundance are indicated in a logarithmic scale (mean values of three independent mRNA datasets). Significance of taxon-specific changes in the SEED level 2 categories is indicated by ^∗^*P* < 0.05, ^∗∗^*P* < 0.01, and ^∗∗∗^*P* < 0.001.

The functional profiles of enriched and taxonomically assigned mRNA were analyzed according to SEED subsystems. The profiles clearly differed between control and NaCl treatments, even at SEED level 1 (**Figure 2C**). The number of categories whose transcript abundance was significantly affected by salt stress changed with SEED level and significance of the *P* and *Q* values (Supplementary Table S3). We first generated functional profiles across all SEED level 1 (Supplementary Figure S4) and 2 categories (Supplementary Figure S5). Based on that, 21 SEED level 2 categories were selected for taxon-specific analysis (**Figure 4** and Supplementary Figure S6). These 21 categories were most significantly affected by salt stress and covered 37.1–49.7% of the enriched mRNA that could be functionally annotated (Supplementary Table S4). On family level, the stress-induced changes in the functional profiles corresponded well to the taxonomic shifts observed for these groups in total mRNA (**Figures 3, 4** and Supplementary Figure S6). This was most evident for the Clostridiaceae, Lachnospiraceae, Methanosarcinaceae, and Methanocellaceae (**Figure 4**). Relative to the other family-level groups, clostridial mRNA was enriched in all the SEED level 2 categories (except for “ABC transporters”). Transcripts affiliated with Lachnospiraceae were enriched in various functional categories at 300 mM NaCl, but not at 600 mM NaCl. Among the Methanosarcinaceae, transcripts involved in “respiration,” “methanogenesis,” “glutamine, glutamate, aspartate, asparagine; ammonia assimilation,” “protein folding,” and “heat shock” were significantly enriched in response to salt stress. Greatest changes in mRNA abundance were related to the categories “respiration” and “methanogenesis.” Their relative transcript level increased from 5.0 ± 0.6% and 13.5 ± 1.1% (control) to 10.1 ± 1.2% and 21.0 ± 1.1% (600 mM NaCl), respectively. The relative transcript levels of all other functional categories either did not change or significantly declined. Transcripts affiliated with Methanocellaceae were underrepresented in all the 21 SEED level 2 categories (**Figure 4**). The mRNA abundance of the Lachnospiraceae and Methanocellaceae was insufficient for a more detailed gene expression analysis. Therefore, subsequent data presentation is only focused on the two most abundant and competitive families: Clostridiaceae and Methanosarcinaceae.

### Expression Response of Particular Pathways, Functions, and Genes

Using Illumina RNA-Seq, no pure culture study has yet been performed for members of the Clostridiaceae and Methanosarcinaceae to assess their transcriptome response to salt stress. Therefore, for these two family-level groups, we expanded our data analysis to the expression of those pathways, functions, and genes that were most responsive to the NaCl treatments (**Tables 1, 2**). This response was driven by the genera *Clostridium* and *Methanosarcina*. Nearly all the mRNA reads assigned to the Clostridiaceae and Methanosarcinaceae were affiliated with these two genera.

**Table 1:** Clostridial transcripts: abundance changes on SEED level 3 and 4^1^.

Level 3/level 4	0 mM	300 mM	600 mM	Log_2_ fold	
				300 mM	600 mM
SSU ribosomal proteins^∗^	0.085	0.317	0.625	1.9	2.9
LSU ribosomal proteins^∗∗^	0.104	0.574	1.036	2.5	3.3
Universal GTPases^∗^	0.027	0.137	0.222	2.3	3.0
Glycolysis and gluconeogenesis^∗∗^	0.020	0.055	0.225	1.5	3.5
Pyruvate:ferredoxin oxidoreductase (PFOR)^∗∗^	0.021	0.107	0.411	2.3	4.3
Acetyl-CoA fermentation to butyrate^∗∗∗^	0.020	0.090	0.350	2.2	4.1
3-hydroxybutyryl-CoA dehydratase	0.002	0.008	0.026	2.0	3.7
Butyryl-CoA dehydrogenase	0.000	0.029	0.036	6.1	6.4
Electron transfer flavoprotein (α)	0.003	0.037	0.239	3.6	6.3
Electron transfer flavoprotein (β)	0.005	0.032	0.073	2.7	3.9
Butanol biosynthesis^∗∗^	0.003	0.004	0.084	0.4	4.8
NADPH-dependent butanol dehydrogenase	0.000	0.003	0.072	2.8	7.4
Lactate fermentation^∗^	0.002	0.005	0.083	1.3	5.4
V-type ATP synthase^∗∗^	0.015	0.051	0.367	1.8	4.6
Na^+^-NQR and Rnf-like group of electron transport complexes^∗∗^	0.004	0.046	0.209	3.5	5.7
Choline, betaine uptake and betaine biosynthesis^∗∗^	0.005	0.028	0.091	2.5	4.2
Glutamine and glutamate biosynthesis^∗^	0.012	0.027	0.056	1.2	2.2
Glutamate synthase (NADPH) large chain	0.005	0.011	0.034	1.1	2.8
Glutamate dehydrogenase	0.002	0.014	0.121	2.8	5.9
Proline, 4-hydroxyproline uptake and utilization^∗∗^	0.001	0.003	0.092	1.6	6.5
Flagellum^∗∗^	0.035	0.110	0.365	1.6	3.4
Flagellar motility^∗^	0.011	0.064	0.312	2.5	4.8
Spore germination^∗^	0.045	0.023	0.012	-1.0	-1.9
Spore coat^∗∗^	0.517	0.228	0.076	-1.2	-2.8

*^1^Percentage and log_*2*_ fold changes in relative transcript abundance affiliated with particular SEED level 3/4 categories are shown in relation to the total mRNA reads that could be functionally annotated on SEED levels 2–4 (Supplementary Table S2). Asterisks indicate different significance levels: ^∗^*P* < 0.05, ^∗∗^*P* < 0.01, ^∗∗∗^*P* < 0.001.*

**Table 2:** Methanosarcinal transcripts: abundance changes on SEED level 3 and 4^1^.

Level 3/level 4	0 mM	300 mM	600 mM	Log_2_ fold	
				300 mM	600 mM
SSU ribosomal proteins^∗∗^	0.470	0.715	1.228	0.6	1.4
LSU ribosomal proteins^∗∗^	0.796	1.184	1.961	0.6	1.3
CODH/ACS^∗∗^	3.510	6.153	8.480	0.8	1.3
Methanogenesis^∗^	9.127	12.698	15.285	0.5	0.7
*mcrA*	2.971	3.613	5.067	0.3	0.8
Thermosome, archaeal^∗∗^	0.127	0.437	0.529	1.8	2.1
Glutamine and glutamate biosynthesis^∗^	0.012	0.018	0.028	0.6	1.2
Glutamate synthase [NADPH] large chain	0.008	0.011	0.024	0.5	1.6
Ammonia assimilation^∗∗∗^	0.026	0.05	0.268	0.9	3.4
Ammonia transporter	0.003	0.025	0.086	3.1	4.8
Nitrogen fixation^∗∗∗^	0.076	0.125	0.357	0.7	2.2
Heat shock DnaK gene cluster extended^∗∗∗^	0.111	0.526	0.716	2.2	2.7

*^1^Percentage and log_*2*_ fold changes in relative transcript abundance affiliated with particular SEED level 3/4 categories are shown in relation to the total mRNA reads that could be functionally annotated on SEED levels 2–4 (Supplementary Table S2). Asterisks indicate different significance levels: ^∗^*P* < 0.05, ^∗∗^*P* < 0.01, ^∗∗∗^*P* < 0.001.*

#### Clostridiaceae (*Clostridium*)

Salt stress induced an up to 3.3 log_2_ fold (600 mM NaCl) increase in the relative expression level of genes involved in transcription and translation, such as those encoding SSU and LSU ribosomal proteins and universal GTPase (**Table 1**). Apparently, Clostridiaceae are able to redirect their energy sources to adapt to environmental stress. The stress-induced stimulation of transcriptional activity and protein biosynthesis was highly significant for gene expression related to the complete set of functional categories required for the generation of energy from sugar fermentation. This included the enrichment of transcripts involved in glycolysis, conversion of pyruvate to acetyl-coenzyme A (acetyl-CoA), and various fermentation pathways (**Table 1**). In particular, we observed a nearly 4.3 log_2_ fold (600 mM NaCl) increase in the abundance of transcripts encoding pyruvate:ferredoxin oxidoreductase (PFOR). This enzyme complex catalyzes a crucial step in the metabolic pathways of most microorganisms: the decarboxylation of pyruvate to form acetyl-CoA. Likewise, clostridial transcripts involved in the fermentation of acetyl-CoA to butyryl-CoA and its further conversion to *n*-butanol increased in abundance with high significance (**Table 1**; Sakuragi et al., 2011). Key enzymes whose transcript abundances greatly increased under salt stress were (i) 3-hydroxybutyryl-CoA dehydratase, (ii) butyryl-CoA dehydrogenase/electron transfer flavoprotein (alpha and beta subunits), and (iii) NADPH-dependent butanol dehydrogenase (log_2_ fold changes of up to 7.4, **Table 1**). In addition, transcripts encoding the fermentation of pyruvate to lactate with its key enzyme lactate dehydrogenase were significantly enriched under salt stress. Apparently, the efflux of Na^+^ was mediated by at least two principal types of ion transporters: V-type ATPases and Na^+^-coupled respiratory enzymes (NADH-quinone oxidoreductases, NQR). Transcripts encoding V-type ATPases and Na^+^-NQR were enriched up to 52-fold (log_2_ fold change of 5.7) in response to salt stress (**Table 1**).

We also observed a stress-induced enrichment of clostridial mRNA involved in “osmotic stress,” primarily related to transcripts encoding ABC transporters for choline, betaine, glycine betaine, proline betaine, carnitine, as well as betaine biosynthesis. These compounds are typical osmoprotectants (Sleator and Hill, 2002; Vyrides et al., 2010). In addition, transcripts involved in “glutamine and glutamate biosynthesis” and “proline, 4-hydroxyproline uptake and utilization” were significantly enriched (**Table 1**). Glutamate, glutamine, and proline are known to act as “compatible solutes.” In particular, the transcript abundance of genes encoding glutamate dehydrogenase and NADPH-dependent glutamate synthase were significantly increased under salt stress. Rice straw has a protein component that accounts for 2–7% (Drake et al., 2002). The stress-induced enrichment of transcripts involved in “osmotic stress” and “proline, 4-hydroxyproline uptake and utilization” relates well to the fact that this protein component is made up of glycine-rich proteins and hydroxyproline-rich glycoproteins (Pan et al., 2011). Thus, the pattern of putative osmoprotectants is similar to that reported for *Bacillus* spp., including the uptake and biosynthesis of proline and/or hydroxyproline preferably at high osmostress (Pittelkow and Bremer, 2011).

Previous studies have shown that in *Clostridium difficile* strain 630, the flagellin gene (*fliC*) will be down-regulated in response to heat shock (Ternan et al., 2012). Contrary to this finding, salt stress triggered a significant increase in the clostridial transcript abundance related to flagellar synthesis (3.4 log_2_ fold) and motility (4.8 log_2_ fold) at 600 mM NaCl (**Table 1**). It is thus tempting to speculate that Clostridiaceae show a chemotactic response to relieve from salt stress by increased cellular motility.

The stress-induced stimulation of clostridial activity is also evidenced by significant decrease in relative transcript abundance of genes involved in spore germination and synthesis of spore coat proteins (**Table 1**). The spore coat represents a proteinaceous shell that encases the spore and plays a major role in spore survival. The -2.8 log_2_ fold (600 mM NaCl) decrease in transcripts involved in the synthesis of spore coat proteins was primarily related to genes encoding “stage IV sporulation protein A (SpoIVA)” and “spore coat protein S” (CotS). SpoIVA is implicated in the coupling of mother cell to forespore gene expression and thus required for spore formation or, more specifically, spore coat localization. The *cotS* gene seems to be required for the assembly of the CotSA protein in spores (McKenney et al., 2013; Putnam et al., 2013).

#### Methanosarcinaceae (*Methanosarcina*)

Overall, the stress-induced enrichment of particular methanosarcinal transcripts in the metatranscriptome was, albeit significant, clearly less pronounced than observed for clostridial transcripts (**Table 2**). Among the enriched methanosarcinal transcripts were those encoding the five-subunit type of the “CO dehydrogenase-acetyl-CoA synthase” (CODH/ACS). Their relative abundance increased from 3.5% (control) to 6.2% (300 mM NaCl) and 8.5% (600 mM NaCl) (**Table 2**). The expression of CODH/ACS, also designated acetyl-CoA decarbonylase synthase (ACDS), is highly indicative of acetoclastic methanogenesis. This complex cleaves acetyl-CoA into a methyl group, an enzyme-bound carbonyl, and free CoA. The methyl group is transferred by CODH/ACS to the C_1_-transferring cofactor tetrahydrosarcinapterin and subsequently reduced to methane (Matschiavelli et al., 2012). Concomitantly, methanosarcinal transcripts encoding methanogenesis were significantly enriched under salt stress. This was primarily related to *mcrA*. In fact, *mcrA* was the most abundant transcript type in enriched mRNA. The increase in methanosarcinal *mcrA* transcript abundance from 2.9% (control) to 3.6% (300 mM) and 5.1% (600 mM NaCl) in the functionally annotated metatranscriptomes corresponds to a decrease in absolute transcript numbers per gram of dry soil by approximately 33% and 59%, respectively (**Table 2** and Supplementary Figure S1). Although results obtained by RT-qPCR and Illumina RNA-Seq are not fully comparable, this correspondence may suggest that relative to the control treatment, most methanosarcinal functions and genes listed in **Table 2** nearly maintained their absolute transcript numbers per gram of dry soil. Genes encoding “heat shock” response and ammonia assimilation may even have increased in absolute transcript numbers (**Table 2**).

The stress-induced enrichment of transcripts related to “heat shock” and encoding chaperones [e.g., Hsp60 (GroEL/GroES) and Hsp70 (DnaJ/DnaK)] was highly significant for the Methanosarcinaceae and clearly more pronounced than for Clostridiaceae (**Figure 4**). Their relative abundance increased from 0.1% to 0.7% (**Table 2**). In particular, we observed at least 3.9-fold enrichment of mRNA linked to Hsp70 (DnaJ/DnaK, Supplementary Table S5). A 2.1 log_2_ fold (600 mM NaCl) increase was also observed for mRNA encoding thermosome subunits (**Table 2**). This is one of the first observations of thermosome gene expression under mesophilic conditions. The thermosome is a class II chaperonin that represents large, barrel-shaped double-ring ATPases. Thermosomes are under study in hyperthermophiles where they are considered crucial for survival (Jia et al., 2015). Our observation may suggest that such class II chaperonins have a broader functional role than previously thought. By contrast, transcripts related to the SEED level 2 category “osmotic stress” were not detected for the Methanosarcinaceae (**Figure 4**). This result is unexpected, given the well-known ability of *Methanosarcina* spp. to produce glycine betaine transporters in response to high salt concentrations (Sowers and Gunsalus, 1995; Roessler et al., 2002). However, transcripts affiliated with the SEED level 3 category “glutamine and glutamate biosynthesis” were significantly enriched under salt stress, in particular those encoding glutamate synthase (NADPH-Gogat; **Table 2**). Apparently, glutamine and/or glutamate were synthesized to act as a compatible solute in these microorganisms. Genes involved in ammonium assimilation showed the greatest stress-induced increase in methanosarcinal transcript level. In particular, transcripts encoding a specific ammonium transporter (AMT) showed a log_2_ fold increase of 4.8 (= 28.7-fold) at 600 mM NaCl. This strong induction of AMT gene expression may be related to a highly increased bioavailability of ammonium upon the addition of NaCl to the paddy soil slurries. Due to cation exchange, the fast release of ammonium bound to soil particles is one of the geochemical key processes that occur in response to increasing salinity (Baldwin et al., 2006; van Dijk et al., 2015).

## Conclusion

This study revealed a significant link between the increase in salinity and community-wide decrease in both mRNA transcription and metabolic activity. mRNA turnover was clearly more responsive to salt stress than rRNA dynamics. The mRNA proportion in total RNA significantly decreased with increasing salinity, from 4.2% (control) to 2.2% (300 mM NaCl) and 1.5% (600 mM NaCl). In short-term responses of up to a few days, the mRNA proportion in total RNA thus may be an excellent proxy for the negative relationship between stress severity and community-wide metabolic activity. On the other hand, the stress-induced decrease in mRNA transcription makes it a particular challenge to obtain a sufficient amount of mRNA for functional metatranscriptome analysis. Alternatively, *rpoB* may be a valuable taxonomic and functional marker for community-wide analysis of stress-induced changes in transcriptional and thus metabolic activity, given that bacterial *rpoB* abundance, diversity, and expression in the environment can be studied by PCR- and RT-PCR-based methods (Case et al., 2007; Vos et al., 2012). A corresponding assay for archaeal *rpoB* has not yet been proposed.

In pure-culture studies, differential gene expression in response to salt stress occurs within minutes. This early-shock response is primarily defined by down-regulation of gene expression. Depending on the ability of the particular microorganism to cope with salt stress, this early period is followed by an adaptive transcriptome response that is governed by up-regulation of gene expression. This adaptive response occurs within a few hours or days (Zaprasis et al., 2015; Nagler et al., 2016). The key taxonomic groups involved in the methanogenic breakdown of plant polymers significantly differed in their ability to cope with salt stress. Presumably, this different ability arises from differences in their genetic potential and metabolic flexibility to reassign available energy resources for cellular adaptation to salt stress. The latter may be directly linked to the metabolic rate with which microorganisms are able to operate in the environment and thus to respond to environmental change. The metabolic rate has been proposed to be a phylogenetically conserved trait, in particular at the family level (Morrissey et al., 2016; Ho et al., 2017).

In order to differentiate the effects of increasing ionic strength (i.e., salinization) from changes in ionic composition (i.e., increasing availability of alternative electron acceptors such as sulfate), salinity was manipulated by NaCl concentration only. Exposed for 7 days to artificial seawater (including 25 mM sulfate), bacterial communities in soil from non-salt-impacted freshwater wetlands showed no significant changes in taxonomic composition (Nelson et al., 2015). Given the rapid turnover of mRNA, the decrease in transcriptional and metabolic activity due to severe salt stress thus can be considered the immediate and primary response that paddy soil microbial communities will show upon seawater intrusion.

## Author Contributions

JP and WL designed the research. JP carried out the laboratory research, while statistical and bioinformatic analyses were performed by JP and CEW. JP and WL wrote the manuscript.

## Conflict of Interest Statement

The authors declare that the research was conducted in the absence of any commercial or financial relationships that could be construed as a potential conflict of interest.
