# Supplementary material for: Short-Term Exposure of Paddy Soil Microbial Communities to Salt Stress Triggers Different Transcriptional Responses of Key Taxonomic Groups
Source: Front Microbiol. 2017 Mar 28;8:400. doi: 10.3389/fmicb.2017.00400 (PMC5368272; doi:10.3389/fmicb.2017.00400)
Supplement: Supplementary file 1 [file Presentation_1.PDF]

SUPPLEMENTARY MATERIAL

Short-term exposure of paddy soil microbial communities to salt stress triggers different transcriptional responses of key taxonomic groups

Jingjing Peng, Carl-Eric Wegner, and Werner Liesack*

Department of Biogeochemistry,
Max Planck Institute for Terrestrial Microbiology,
Marburg, Germany

The supplementary material contains
6 figures, 5 tables, and 12 pages in total.

*Corresponding author:
Werner Liesack, Max Planck Institute for Terrestrial Microbiology, Karl-von-Frisch-Str. 10, 35043 Marburg, Germany; e-mail: liesack@mpi-marburg.mpg.de

[#]Present address

Friedrich Schiller University Jena, Institute of Ecology, Aquatic Geomicrobiology,

Dornburger Str. 159, 07749 Jena, Germany

Supplementary Figures

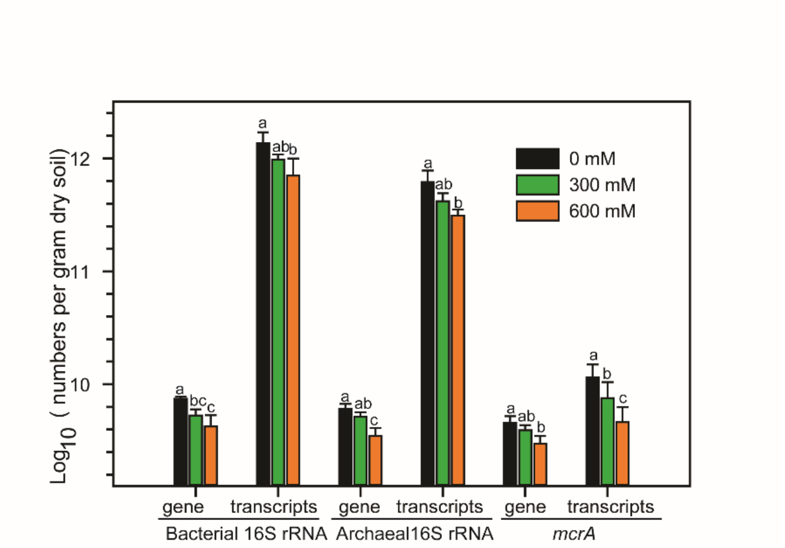

Figure S1. Bacterial and archaeal 16S rRNA gene and transcript numbers per gram of dry soil, and those of *mcrA* (means±SE; n=3). Different letters (a, b, c) indicate a significant difference in gene or transcript number among the three treatments (P<0.05).

	rRNA 0 mM	rRNA 300 mM	rRNA 600 mM	mRNA 0 mM	mRNA 300 mM	mRNA 600 mM
Firmicutes	41.11	38.66	34.8	37.5	41.61	33.21
Euryarchaeota	17.99	20.32	19.69	35.78	36.12	44.04
Proteobacteria	14.31	13.5	15.73	14.68	17.06	16.44
Actinobacteria	8.61	11.45	11.59	2.72	1.59	2.4
Chloroflexi	3.84	3.92	4.58	1.3	0.9	1.06
Acidobacteria	2.98	2.26	2.49	1.87	0.19	0.43
Planctomycetes	3.36	3.06	3.78	0.96	0.29	0.47
Others	6.63	5.9	6.22	4.36	1.73	1.36
Unclassified	1.18	0.94	1.14	0.83	0.52	0.6
Clostridiaceae	20.66	21.36	22.32	8.72	13.48	19.81
Lachnospiraceae	0.92	1.81	0.9	2.29	6.02	2.87
Ruminococcaceae	3.22	2.47	1.44	4.2	4.02	1.81
Bacillaceae	2.98	2.9	2.61	3.31	3.02	2.75
Peptococcaceae	1.79	1.83	1.53	3.28	2.18	0.97
Syntrophomonadaceae	0.31	0.09	0.08	1.09	0.28	0
Veillonellaceae	0.32	0.45	0.31	1.22	0.58	0.27
Anaeromyxobacteraceae	1.05	0.95	1.1	0.31	0	0
Methanosarcinaceae	15.92	18.41	17.72	37.62	43.87	47.87
Methanosaetaceae	0.33	0.36	0.41	1.06	0.47	1.2
Methanocellaceae	1.11	0.73	0.82	7.36	1.25	1.46
Methanobacteriaceae	0.17	0.24	0.26	0.56	1.37	1.14
Others	27.45	26.78	27.14	25.78	21.79	17.88
Unclassified	23.79	21.63	23.36	3.21	1.68	1.97

0 : 0.6
0.6 : 1.5
1.5 : 3.2
3.2 : 17.8
17.8 : 47.9

Figure S2. Phylum- and family-level changes in relative abundance: (a) 16S rRNA and (b) enriched mRNA: 0 mM (control), 300 mM NaCl, and 600 mM NaCl. Percentage values are shown for those phyla and families that were most abundant (>1%) in 16S rRNA analysis (Figure 3, Supplementary Figure S3).

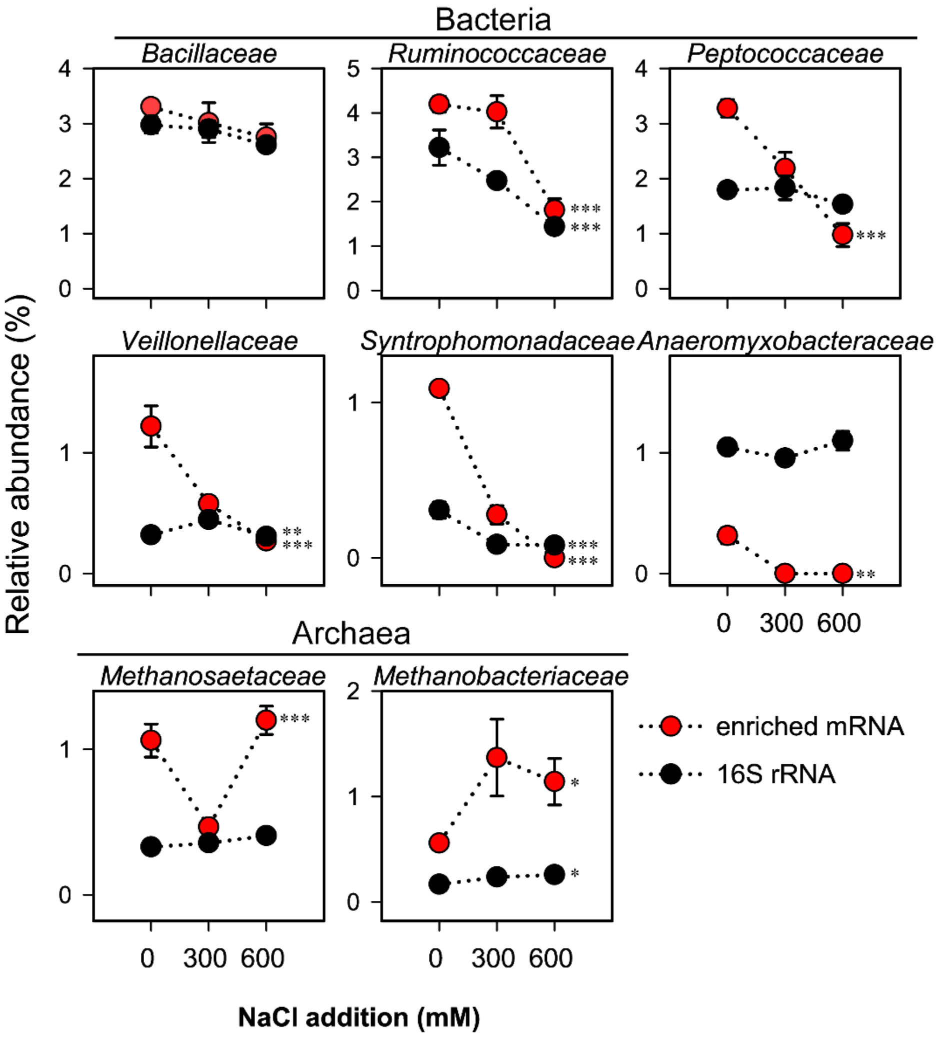

Figure S3. Taxon-specific changes on rRNA and mRNA levels in response to the different NaCl treatments. In correspondence to the key taxonomic groups (Figure 3), stress-induced changes are shown for the next-abundant bacterial and archaeal groups on family level (means±SE; n=3). See Supplementary Tables S1 and S2 for sequencing statistics of 16S rRNA and taxonomically assigned mRNA, respectively. Broken lines are added only for illustrative purposes. Low-abundant populations are not shown (<1%). Significant changes in relative abundance are indicated by * ($P<0.05$), ** ($P<0.01$), and *** ($P<0.001$).

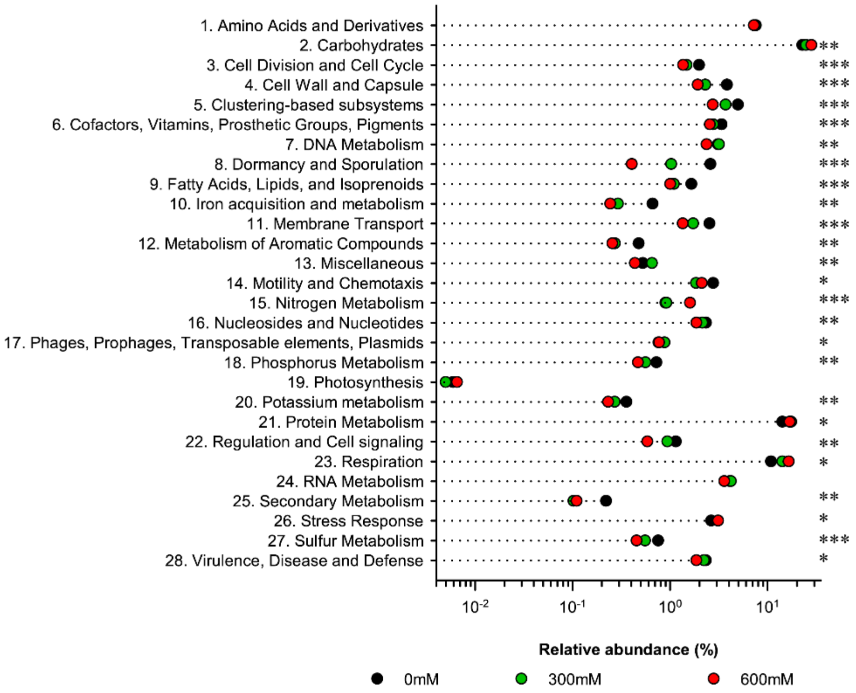

Figure S4. Stress-induced changes in enriched mRNA affiliated with SEED level 1 categories. Note that relative abundance changes are indicated in a logarithmic scale (mean values of three independent mRNA datasets for each treatment). Significant changes in relative abundance are indicated by * (*P*<0.05), ** (*P* <0.01), and *** (*P* <0.001).

Relative to the control, the mRNA abundance of most SEED level 1 categories significantly decreased in response to salt stress. One of the most striking examples is ‘dormancy and sporulation’. However, mRNA affiliated with ‘carbohydrates’ (22.7% to 28.1%), ‘respiration’ (10.8% to 16.4%), and ‘nitrogen metabolism’ (0.9% to 1.6%) was significantly enriched under salt stress. ‘Carbohydrates’ and ‘respiration’ showed greatest mRNA abundances among all SEED level 1 categories. In addition, mRNA affiliated with ‘protein metabolism’ and ‘stress response’ increased by trend in transcript abundance, relative to the other SEED level 1 categories.

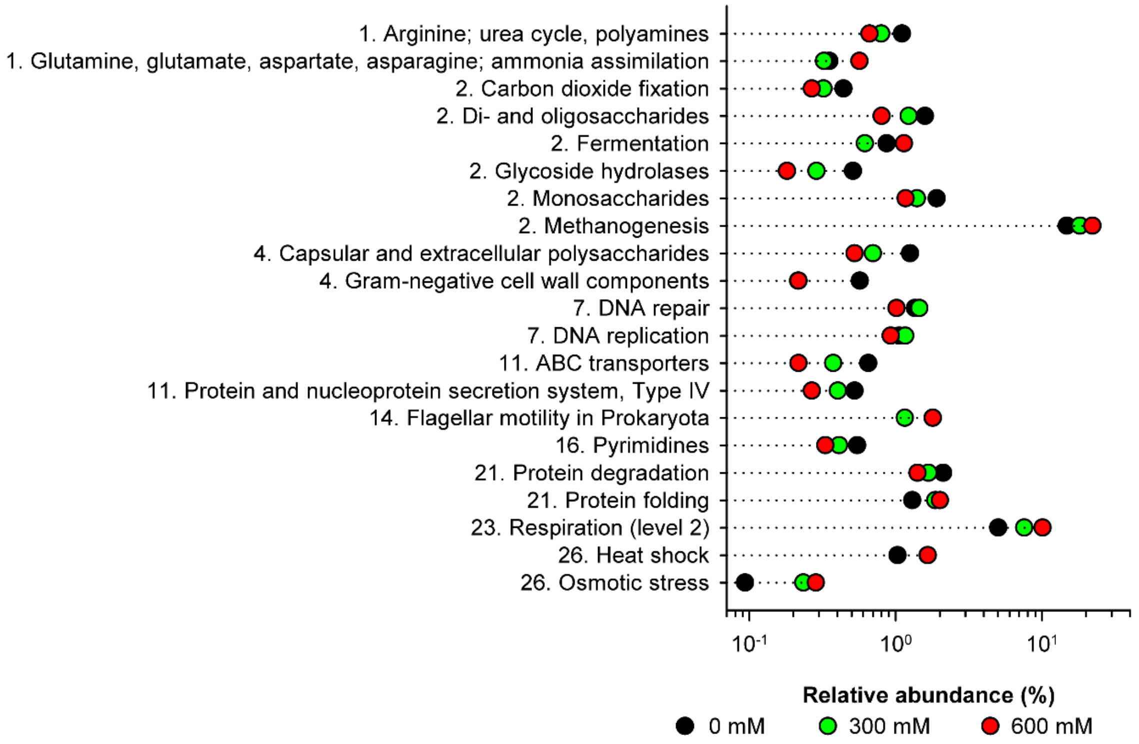

Figure S5. Stress-induced changes in enriched mRNA affiliated with 21 SEED level 2 categories. Note that relative abundance changes are indicated in a logarithmic scale (mean values of three independent mRNA datasets for each treatment). These 21 categories were most significantly affected by salt stress (*P*<0.01) in community-wide gene expression. Numbering of categories relates to the SEED level 1 categories as shown in Supplementary Figure S4.

Our data analysis focused on SEED level 2 categories that met the following criteria: (i) contribution of ≥ 0.5% to the functionally annotated metatranscriptomes in at least one of the experimental treatments and (ii) significantly affected by salt stress with *P* value < 0.01 and false discovery rate (FDR)-adjusted *Q* value < 0.05. As one exception, 'flagellar motility in prokaryota' was included in this data analysis. An increase in relative transcript abundance was observed for the following categories (level 1 categories shown in parenthesis): 'one-carbon metabolism' (carbohydrates); 'respiration' (respiration); 'protein folding' (protein metabolism); 'heat shock' and 'osmotic stress' (stress response). In particular, the transcript abundance of 'one-carbon metabolism' increased from 14.8% (control) to 22.2% (600 mM NaCl).

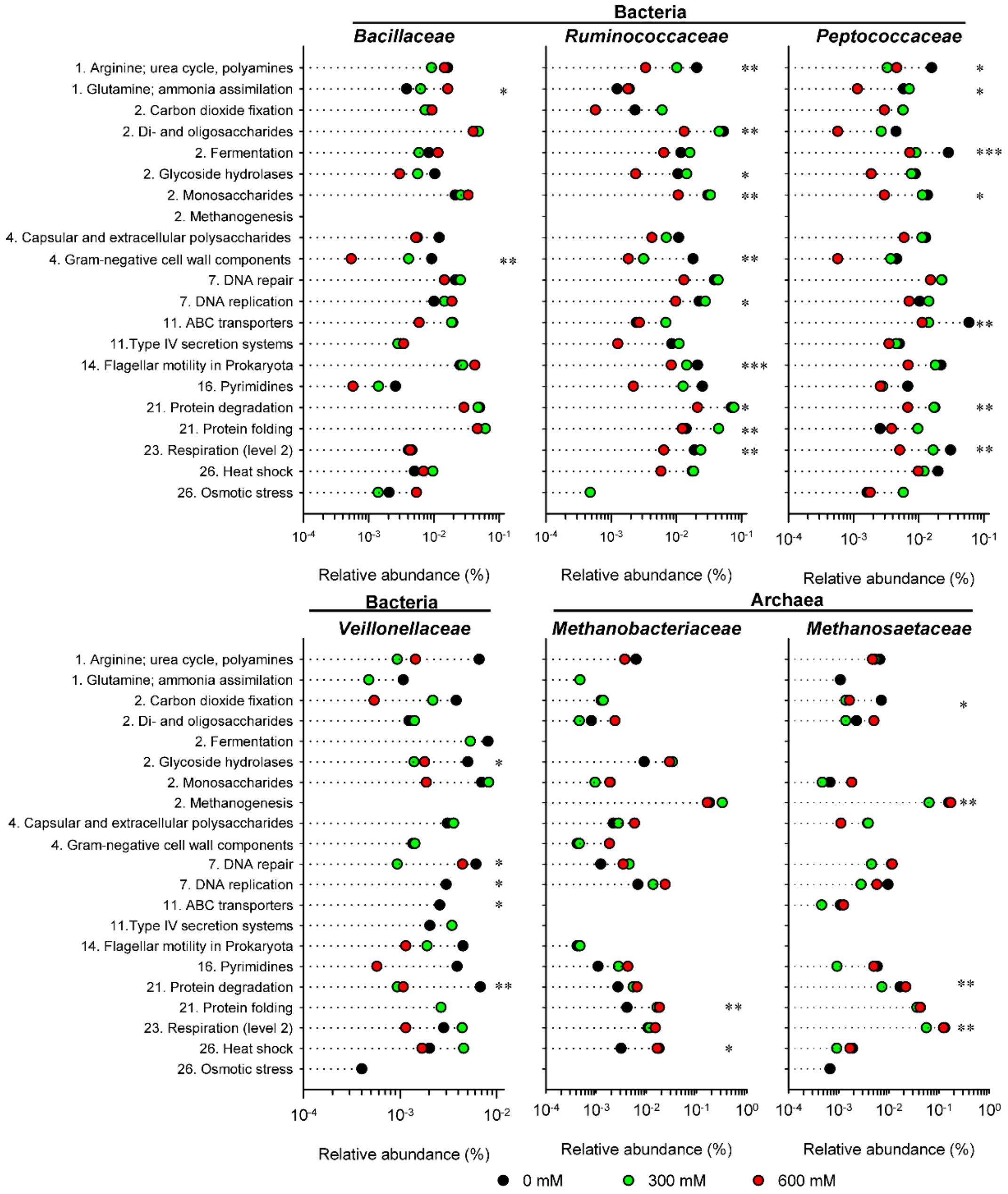

Figure S6. Taxon-specific changes in 21 SEED level 2 categories (Supplementary Figure S5). In correspondence to the key taxonomic groups (Figure 4), percentage changes in relative transcript abundance are shown for the six next-abundant family-level groups in relation to the total mRNA reads that could be assigned on SEED levels 2-4 (Supplementary Table S2). Numbering of categories relates to the SEED level 1 categories as shown in Supplementary Figure S4. Note that changes in relative mRNA abundance are indicated in a logarithmic scale (mean values of three independent mRNA datasets). Significance of taxon-specific changes in the SEED level 2 categories is indicated by * (P<0.05), ** (P<0.01), and *** (P<0.001).

Supplementary Tables

Table S1. Sequencing statistics of bacterial and archaeal 16S rRNA in metatranscriptomic analysis of total RNA.

Treatment (Replicate)[1]	0 mM) *Rep. 1*	0 mM *Rep. 2*	0 mM *Rep. 3*	300 mM *Rep. 1*	300 mM *Rep. 2*	300 mM) *Rep. 3*	600 mM *Rep. 1*	600 mM *Rep. 2*	600 mM *Rep. 3*	Total
**Processed reads[2]**	536,036	823,258	711,801	715,710	530,576	751,931	714,063	635,986	574,282	5,993,643
**mRNA[3]**	16,041 (2.99)	39,677 (4.82)	34,534 (4.85)	17,097 (2.39)	11,092 (2.09)	14,745 (1.96)	11,688 (1.64)	9,060 (1.42)	8,932 (1.56)	162,866
**16S rRNA[4]**	295,624 (55.15)	432,210 (52.5)	373,695 (52.5)	353,489 (49.39)	275,740 (51.97)	393,109 (52.28)	358,459 (50.2)	325,688 (51.21)	290,816 (50.64)	3,098,833
**Assigned on phylum level[5]**	285,660 (96.63)	415,098 (96.04)	359,220 (96.13)	343,097 (97.06)	268,838 (97.50)	382,468 (97.30)	348,891 (97.33)	316,904 (97.30)	282,521 (97.15)	3,002,697
**Assigned on family level[6]**	251,365 (85.03)	364,632 (84.36)	315,948 (84.54)	305,675 (86.47)	241,576 (87.61)	342,145 (87.04)	310,694 (86.67)	280,512 (86.13)	247,394 (85.07)	2,659,941

[1] Metatranscriptomic analysis of total RNA was performed for the control (0 mM NaCl) and for half- and full-strength seawater salinity (300 mM and 600 mM NaCl). Sequencing statistics is shown for each replicate treatment separately.

[2] Total number of quality-filtered Illumina RNA-Seq reads of 240-250 bp length.

[3] Total number of mRNA reads obtained by elimination of rRNA and non-coding small RNA via SORTMERNA 2.0. Numbers in parenthesis indicate the percentage of processed reads.

[4] Total number of bacterial and archaeal 16S rRNA reads extracted by SORTMERNA 2.0. Numbers in parenthesis indicate the percentage of processed reads.

[5,6] Total number of bacterial and archaeal 16S rRNA reads that could be assigned on phylum and family levels. Numbers in parenthesis indicate the percentage of total 16S rRNA reads that could be assigned on phylum or family level.

Table S2. Sequencing statistics of mRNA in metatranscriptomic analysis of enriched mRNA.

**Treatment (replicate)[1]**	**0 mM** *Rep. 1*	**0 mM** *Rep. 2*	**0 mM** *Rep. 3*	**300 mM** *Rep. 1*	**300 mM** *Rep. 2*	**300 mM** *Rep. 3*	**600 mM** *Rep. 1*	**600 mM** *Rep. 2*	**600 mM** *Rep. 3*	**Total**
**Processed reads[2]**	1,007,536	877,185	905,685	672,541	618,463	903,240	535,772	933,024	775,099	7,228,545
**Reads with NCBI nr hit[3]**	256,664 (25.47)	175,888 (20.05)	269,042 (29.71)	71,421 (10.62)	182,839 (29.56)	209,913 (23.24)	118,261 (22.07)	167,703 (17.97)	156,178 (20.15)	1,607,909
**Assigned on domain level[4]**	224,139 (87.33)	145,325 (82.62)	233,225 (86.69)	54,370 (76.13)	162,190 (88.71)	179,707 (85.61)	101,283 (85.64)	139,555 (83.22)	129,409 (82.86)	1,369,203
**Assinged on phylum level[5]**	193,478 (75.38)	121,589 (69.13)	199,495 (74.15)	46,392 (64.96)	141,464 (77.37)	153,973 (73.35)	89,681 (75.83)	125,132 (74.62)	114,002 (72.99)	1,185,206
**Assigned on family level[6]**	134,452 (52.38)	82,050 (46.65)	135,798 (50.47)	32,598 (45.64)	99,410 (54.37)	104,444 (49.76)	67,758 (57.30)	99,153 (59.12)	86,523 (55.40)	842,186
**SEED level 1 subsystem[7]**	95,057 (37.04)	58,830 (33.45)	98,993 (36.79)	22,430 (31.41)	84,380 (46.15)	82,063 (39.09)	53,302 (45.07)	70,475 (42.02)	66,716 (42.72)	632,246
**SEED level 2-4 subsystems[8]**	79,881 (31.12)	49,087 (27.91)	83,087 (30.88)	19,299 (27.02)	72,298 (39.54)	70,055 (33.37)	46,131 (39.01)	61,699 (36.79)	58,066 (37.18)	539,603

[1] Sequencing statistics is shown for each replicate treatment separately.

[2] Total number of quality-filtered Illumina RNA-Seq reads of 240-250 bp length.

[3] Number of mRNA reads that had a homolog in NCBI nr database using BLASTx (*e*-value cutoff of $1e^{-5}$). mRNA reads with NCBI nr hits were subjected to taxonomic assignment and functional annotation. Numbers in parenthesis indicate percentage of quality-filtered reads that had a NCBI nr hit.

[4, 5, 6] Number of mRNA reads that could be assigned on domain, phylum, or family level to a taxonomic group by MEGAN LCA, using the BLASTx output file. Numbers in parenthesis indicate the percentage of mRNA reads with NCBI nr hit that could be taxonomically assigned on domain, phylum, or family level.

[7, 8] Number of mRNA reads that could be functionally annotated on SEED level 1 or levels 2-4 subsystems using MEGAN5. Numbers in parenthesis indicate the percentage of mRNA-tags with NCBI nr hit that could be assigned to a functional category in SEED level 1 or levels 2-4 subsystems.

Table S3. Significance statistics for SEED levels 1, 2, 3, and 4[1].

	P<0.05	P<0.01	P<0.001	Q<0.1	Q<0.05	Q<0.01
**Level 1**	22	18	8	22	21	16
**Level 2**	76	56	30	84	69	41
**Level 3**	243	149	53	238	183	55
**Level 4**	954	456	151	480	262	44

[1]The table sums up the number of categories on SEED levels 1, 2, 3, and 4 (gene function) that depending on the *P* and *Q* values, exhibited significant changes between the NaCl treatments. If not particularly mentioned, tests were considered significant if they had at least a *P*-value < 0.05 and a *Q*-value < 0.1.

Table S4. Number of mRNA reads grouped into particular functional categories on SEED level 2.

Index	Level 1[1]	Level 2[1]	0 mM *Rep. 1*	0 mM *Rep. 2*	0 mM *Rep. 3*	300 mM *Rep. 1*	300 mM *Rep. 2*	300 mM *Rep. 3*	600 mM *Rep. 1*	600 mM *Rep. 2*	600 mM *Rep. 3*
1	**Amino Acids and Derivatives**	**Arginine; urea cycle, polyamines**	821 (1.03)	584 (1.19)	910 (1.10)	124 (0.64)	633 (0.88)	615 (0.88)	301 (0.65)	376 (0.61)	424 (0.73)
1	**Amino Acids and Derivatives**	**Glutamine glutamate aspartate asparagine ammonia assimilation**	279 (0.35)	179 (0.36)	286 (0.34)	55 (0.28)	225 (0.31)	262 (0.37)	254 (0.55)	378 (0.61)	311 (0.54)
2	**Carbohydrates**	**CO_2 fixation**	354 (0.44)	220 (0.45)	357 (0.43)	63 (0.33)	224 (0.31)	231 (0.33)	117 (0.25)	145 (0.24)	182 (0.31)
2	**Carbohydrates**	**Di- and oligosaccharides**	1,218 (1.52)	784 (1.60)	1,365 (1.64)	213 (1.10)	893 (1.24)	941 (1.34)	399 (0.86)	388 (0.63)	532 (0.92)
2	**Carbohydrates**	**Fermentation**	648 (0.81)	484 (0.99)	675 (0.81)	106 (0.55)	454 (0.63)	471 (0.67)	527 (1.14)	715 (1.16)	654 (1.13)
2	**Carbohydrates**	**Glycoside hydrolases**	425 (0.53)	241 (0.49)	425 (0.51)	49 (0.25)	209 (0.29)	224 (0.32)	91 (0.20)	101 (0.16)	106 (0.18)
2	**Carbohydrates**	**Monosaccharides**	1,480 (1.85)	948 (1.93)	1,615 (1.94)	238 (1.23)	1,006 (1.39)	1,096 (1.56)	555 (1.20)	675 (1.09)	703 (1.21)
2	**Carbohydrates**	**One-carbon metabolism/Methanogenesis**	12,466 (15.61)	6,511 (13.22)	12,859 (15.48)	4,055 (21.01)	12,310 (17.03)	11,587 (16.54)	9,745 (21.12)	13,389 (21.70)	13,725 (23.64)
4	**Cell Wall and Capsule**	**Capsular and extracellular polysaccharides**	1,022 (1.28)	565 (1.15)	1,106 (1.33)	116 (0.60)	518 (0.72)	550 (0.79)	259 (0.56)	293 (0.47)	311 (0.54)
4	**Cell Wall and Capsule**	**Gram-negative cell wall components**	477 (0.60)	255 (0.52)	494 (0.59)	37 (0.19)	160 (0.22)	164 (0.23)	110 (0.24)	89 (0.14)	156 (0.27)
7	**DNA Metabolism**	**DNA repair**	1,024 (1.28)	644 (1.31)	1,219 (1.47)	268 (1.39)	996 (1.38)	1,118 (1.60)	519 (1.13)	542 (0.88)	604 (1.04)
7	**DNA Metabolism**	**DNA replication**	794 (0.99)	519 (1.06)	936 (1.13)	219 (1.13)	852 (1.18)	827 (1.18)	463 (1.00)	540 (0.88)	521 (0.90)
11	**Membrane Transport**	**ABC transporters**	513 (0.64)	346 (0.70)	501 (0.60)	68 (0.35)	280 (0.39)	270 (0.39)	98 (0.21)	121 (0.20)	141 (0.24)
11	**Membrane Transport**	**Protein and nucleoprotein secretion system Type IV**	396 (0.50)	256 (0.52)	462 (0.56)	76 (0.39)	281 (0.39)	298 (0.43)	157 (0.34)	128 (0.21)	148 (0.25)
14	**Motility and Chemotaxis**	**Flagellar motility in Prokaryota**	1,353 (1.69)	981 (2.00)	1,397 (1.68)	163 (0.84)	927 (1.28)	942 (1.34)	780 (1.69)	1,413 (2.29)	823 (1.42)
16	**Nucleosides and Nucleotides**	**Pyrimidines**	426 (0.53)	274 (0.56)	454 (0.55)	81 (0.42)	287 (0.40)	292 (0.42)	147 (0.32)	191 (0.31)	212 (0.37)
21	**Protein Metabolism**	**Protein degradation**	1,637 (2.05)	1,148 (2.34)	1,624 (1.95)	321 (1.66)	1,184 (1.64)	1,210 (1.73)	672 (1.46)	857 (1.39)	805 (1.39)
21	**Protein Metabolism**	**Protein folding**	1,064 (1.33)	672 (1.37)	1,001 (1.20)	394 (2.04)	1,382 (1.91)	1,156 (1.65)	1,017 (2.20)	1,124 (1.82)	1,164 (2.00)
23	**Respiration**	**Respiration (level 2)**	4,404 (5.51)	2,057 (4.19)	4,485 (5.40)	1,599 (8.29)	5,726 (7.92)	4,546 (6.49)	3,888 (8.43)	6,854 (11.11)	6,244 (10.75)
26	**Stress Response**	**Heat shock**	809 (1.01)	514 (1.05)	862 (1.04)	321 (1.66)	1,169 (1.62)	1,183 (1.69)	864 (1.87)	915 (1.48)	940 (1.62)
26	**Stress Response**	**Osmotic stress**	75 (0.09)	41 (0.08)	86 (0.10)	49 (0.25)	141 (0.20)	178 (0.25)	149 (0.32)	190 (0.31)	131 (0.23)
		**Sum of mRNA reads listed above[2]**	31,685 (39.67)	18,223 (37.12)	33,119 (39.86)	8,615 (44.64)	29,857 (41.30)	28,161 (40.20)	21,112 (45.77)	29,424(47.69)	28,837 (49.66)
		**Total number of mRNA reads[3]**	79,881 (100)	49,087 (100)	83,087 (100)	19,299 (100)	72,298 (100)	70,055 (100)	46,131 (100)	61,699 (100)	58,066 (100)

[1] SEED level 1 and 2 categories, respectively.

[2] Sum of mRNA reads grouped into those SEED level 2 categories listed above for each replicate treatment.

[3] Total number of mRNA reads that could be functionally annotated for each replicate treatment on SEED level 2.

Table S5. Changes in the relative expression level of genes (SEED level 4 categories) involved in stress response.

		Total					*Clostridiaceae*					*Methanosarcinaceae*				
**L2[1]**	**L4[1]**	***Q*-values[2]**	***P*-values[2]**	**0 mM[3]**	**300 mM[4]**	**600 mM[5]**	***Q*-values[2]**	***P*-values[2]**	**0 mM[3]**	**300 mM[4]**	**600 mM[5]**	***Q*-values[2]**	***P*-values[2]**	**0 mM[3]**	**300 mM[4]**	**600 mM[5]**
**Heat shock**	**Chaperone protein DnaJ**	0.044	0.002	0.121	0.241	0.273	0.216	0.034	0.001	0.010	0.010	0.134	0.021	0.024	0.097	0.119
**Heat shock**	**Chaperone protein DnaK**	0.020	0.000	0.482	0.829	0.918	0.048	0.001	0.010	0.024	0.044	0.005	0.000	0.061	0.290	0.419
**Heat shock**	**Heat shock protein GrpE**	0.030	0.001	0.050	0.185	0.206	0.342	0.092	0.000	0.005	0.005	0.033	0.002	0.014	0.118	0.166
**Heat shock**	**Signal peptidase-like protein**	0.093	0.010	0.027	0.044	0.020	0.408	0.129	0.004	0.011	0.007	--	--	--	--	--
**Osmotic stress**	**Choline ABC transport system, ATP-binding protein OpuBA**	0.005	0.000	0.001	0.015	0.048	0.010	0.000	0.000	0.000	0.004	--	--	--	--	--
**Osmotic stress**	**Choline dehydrogenase (EC 1.1.99.1)**	0.197	0.040	0.012	0.003	0.002	--	--	--	--	--	--	--	--	--	--
**Osmotic stress**	**Choline-sulfatase (EC 3.1.6.6)**	0.178	0.034	0.018	0.008	0.005	0.769	0.422	0.001	0.000	0.000	--	--	--	--	--
**Osmotic stress**	**Glycine betaine ABC transport system, glycine betaine-binding protein OpuAC**	0.093	0.010	0.002	0.029	0.042	0.135	0.012	0.000	0.001	0.014	--	--	--	--	--
**Osmotic stress**	**Glycine betaine ABC transport system, permease protein OpuAB**	0.149	0.024	0.000	0.009	0.002	0.408	0.129	0.000	0.001	0.000	--	--	--	--	--
**Osmotic stress**	**Glycine betaine transporter OpuD**	0.175	0.033	0.001	0.004	0.024	0.218	0.035	0.000	0.000	0.021	--	--	--	--	--
**Osmotic stress**	**High-affinity choline uptake protein BetT**	0.113	0.014	0.000	0.001	0.005	0.642	0.422	0.000	0.000	0.001	--	--	--	--	--
**Osmotic stress**	**Osmotically activated L-carnitine/choline ABC transporter, ATP-binding protein OpuCA**	0.171	0.031	0.003	0.019	0.018	0.270	0.052	0.000	0.001	0.007	--	--	--	--	--
**Osmotic stress**	**Osmotically activated L-carnitine/choline ABC transporter, permease protein OpuCB**	0.053	0.003	0.003	0.053	0.022	0.004	0.000	0.000	0.000	0.008	--	--	--	--	--
**Osmotic stress**	**Osmotically activated L-carnitine/choline ABC transporter, substrate-binding protein OpuCC**	0.204	0.042	0.002	0.004	0.043	0.575	0.422	0.000	0.000	0.000	--	--	--	--	--
**Oxidative stress**	**Alkyl hydroperoxide reductase subunit C-like protein**	0.029	0.001	0.169	0.065	0.065	0.511	0.183	0.003	0.005	0.001	0.374	0.128	0.004	0.008	0.005
**Oxidative stress**	**Peroxide stress regulator**	0.139	0.022	0.002	0.010	0.002	0.692	0.340	0.000	0.001	0.001	--	--	--	--	--
**Oxidative stress**	**Rubrerythrin**	0.213	0.046	0.474	0.414	0.295	0.245	0.043	0.050	0.068	0.118	0.479	0.201	0.052	0.059	0.028
**Oxidative stress**	**Superoxide dismutase [Cu-Zn] precursor (EC 1.15.1.1)**	0.045	0.002	0.056	0.016	0.004	0.163	0.018	0.002	0.000	0.000	--	--	--	--	--
**Oxidative stress**	**Superoxide dismutase [Fe] (EC 1.15.1.1)**	0.084	0.008	0.029	0.007	0.008	0.975	0.968	0.000	0.000	0.001	--	--	--	--	--
**Oxidative stress**	**Superoxide dismutase [Mn/Fe] (EC 1.15.1.1)**	0.662	0.043	0.033	0.037	0.013	--	--	--	--	--	0.253	0.065	0.019	0.023	0.008
**Oxidative stress**	**Superoxide dismutase [Mn] (EC 1.15.1.1)**	0.205	0.002	0.012	0.005	0.003	--	--	--	--	--					
**Oxidative stress**	**Superoxide reductase (EC 1.15.1.2)**	0.368	0.129	0.026	0.054	0.038	0.212	0.032	0.004	0.011	0.022	0.707	0.540	0.002	0.003	0.001
**Oxidative stress**	**Rubredoxin**	0.204	0.043	0.040	0.041	0.022	0.689	0.422	0.000	0.000	0.000	0.667	0.330	0.009	0.012	0.007
**Oxidative stress**	**Rubredoxin-oxygen oxidoreductase**	0.149	0.024	0.015	0.021	0.012	0.676	0.559	0.000	0.002	0.001	0.548	0.242	0.005	0.007	0.004

[1]Percentage values of mRNA abundance affiliated with the different SEED level 4 categories are shown for each experimental treatment in relation to the total mRNA reads that could be functionally annotated on SEED levels 2-4 (Supplementary Table S2).

[2] *Q*-values were calculated by STAMP (ANOVA) with corrections for multiple tests. Corrections were adjusted to produce a final Benjamini and Hochberg false discovery rate. *P*-values were calculated by STAMP (ANOVA) without correction.

[3, 4, 5] Mean percentage values of three independent datasets obtained each for control (0 mM) and the salt treatments (300 mM and 600 mM NaCl).
